# Determination of Intensity-Based Stochastic Models for Terrestrial Laser Scanners Utilising 3D-Point Clouds

**DOI:** 10.3390/s18072187

**Published:** 2018-07-07

**Authors:** Daniel Wujanz, Mathias Burger, Felix Tschirschwitz, Tassilo Nietzschmann, Frank Neitzel, Thomas P. Kersten

**Affiliations:** 1 Institute of Geodesy and Geoinformation Science, Technische Universität Berlin, 10623 Berlin, Germany; mathias.burger@tu-berlin.de (M.B.); tassilo.a.n@gmail.com (T.N.); frank.neitzel@tu-berlin.de (F.N.); 2 Photogrammetry & Laser Scanning Lab, HafenCity University Hamburg, 20457 Hamburg, Germany; felix.tschirschwitz@hcu-hamburg.de (F.T.); thomas.kersten@hcu-hamburg.de (T.P.K.)

**Keywords:** individual point quality, precision, rangefinder, stochastic modelling, terrestrial laser scanning

## Abstract

Recent advances in stochastic modelling of reflectorless rangefinders revealed an inherent relationship among raw intensity values and the corresponding precision of observed distances. In order to derive the stochastic properties of a terrestrial laser scanner’s (TLS) rangefinder, distances have to be observed repeatedly. For this, the TLS of interest has to be operated in the so-called 1D-mode—a functionality which is offered only by a few manufacturers due to laser safety regulations. The article at hand proposes two methodologies to compute intensity-based stochastic models based on capturing geometric primitives in form of planar shapes utilising 3D-point clouds. At first the procedures are applied to a phase-based Zoller + Fröhlich IMAGER 5006h. The generated results are then evaluated by comparing the outcome to the parameters of a stochastic model which has been derived by means of measurements captured in 1D-mode. Another open research question is if intensity-based stochastic models are applicable for other rangefinder types. Therefore, one of the suggested procedures is applied to a Riegl VZ-400i impulse scanner, as well as a Leica ScanStation P40 TLS that deploys a hybrid rangefinder technology. The generated results successfully demonstrate alternative methods for the computation of intensity-based stochastic models as well as their transferability to other rangefinder technologies.

## 1. Introduction

A core competence of geodesists is the profound knowledge of the stochastic properties of the measuring sensors used. This knowledge is essential to understand the influence of individual observations during parameter estimation, to detect outliers or to separate statistically significant deformations from the measurement uncertainty. TLS is currently playing a strong role in a wide range of applications including stability assessment [1], bridge asset management [2], and automatic computational model generation for historic buildings [3]. However, the core competence mentioned at the beginning was ignored, since no stochastic model was available for more than a decade—neither from the manufacturers, nor from the side of academia. This circumstance is particularly surprising as an appropriate stochastic model is of vital importance for many important applications, e.g., the calibration of TLS [4], the registration of point clouds [5,6] or direct georeferencing of TLS [7,8].

The frequently-used assumption of identical precision for all captured 3D points of a laser scan has already been disproved by several authors, e.g., by Schaer et al. [9]. One of the first publications dealing with the analysis of systematic and random object influences on TLS measurements comes from Böhler et al. [10], who showed a relationship between the reflectivity of the surface of the measured object and the noise of the measured distance. Elkhrachy and Niemeier [11] introduced a distance-dependent stochastic model in the adjustment of transformation parameters. Several authors [12,13,14,15] performed studies on the influence of surface properties on the noise of distance measurements which were observed with terrestrial laser scanners. Soudarissanane et al. [16] and Zámečníková et al. [17] investigated the relationship between the incidence angle of the signal on the object surface and the absolute and relative accuracy of the distance measurement.

All these contributions were undeniably of great scientific importance, since they show individual aspects of the measurement uncertainty for individual points. However, it is not possible to establish the functional relationship for an all-embracing stochastic model considering all the aforementioned influencing factors. Even if this were possible, the necessary radiometric properties of the object surfaces are usually not known and would have to be modelled separately. Current research contributions by Wujanz et al. [18] show an alternative approach in which the signal strength of the reflected measurement signal and the noise of the distance measurement are linked to derive a stochastic model. The validity of such an intensity-based stochastic model has been demonstrated in several investigations by Wujanz et al. [18,19] and was consequently used for various purposes by Cefalu et al. [20] and Xu et al. [21]. Ozendi et al. [22] correctly stated that intensity-based stochastic models can only be used where raw intensity values in the measurement data can be accessed. Therefore, it is somewhat surprising that their own approach requires radiometric information and, consequently, is subject to the same restriction.

The reason why raw intensity values are suitable for stochastic modelling of reflectorless rangefinders can be justified by the radar range equation:

(1) $$P_{r}=\frac{P_{e}D_{r}^{2}\rho_{\lambda }\cos\alpha }{4R^{2}}\eta_{system}\eta_{atmos}$$

as given, e.g., by Wagner et al. [23], which is not only valid for microwaves, but also for electro-magnetic signals with shorter wavelengths as explained by Höfle and Pfeifer [24]. The parameters *P_e_* and *P_r_* denote the power of the emitted and received signals, respectively. The scanner’s receiver aperture diameter is represented by *D_r_* while *η_system_* signifies its transmission coefficient. Atmospheric influences are denoted by *η_atmos_* whereas the range between the scanner and object point is symbolised by *R*. The angle of incidence is represented by *α*, the quotient of reflection of the object’s surface in the wavelength of the scanner is described by *ρ_λ_*. In summary, all parameters in the equation have an immediate impact onto the received signal strength *P_r_*. From another perspective it can be concluded that all relevant parameters in (1) are inherently considered in *P_r_* which makes individual consideration of the influencing factors unnecessary and hence justifies the intensity-based method suggested by Wujanz et al. [18].

**Figure 1:**
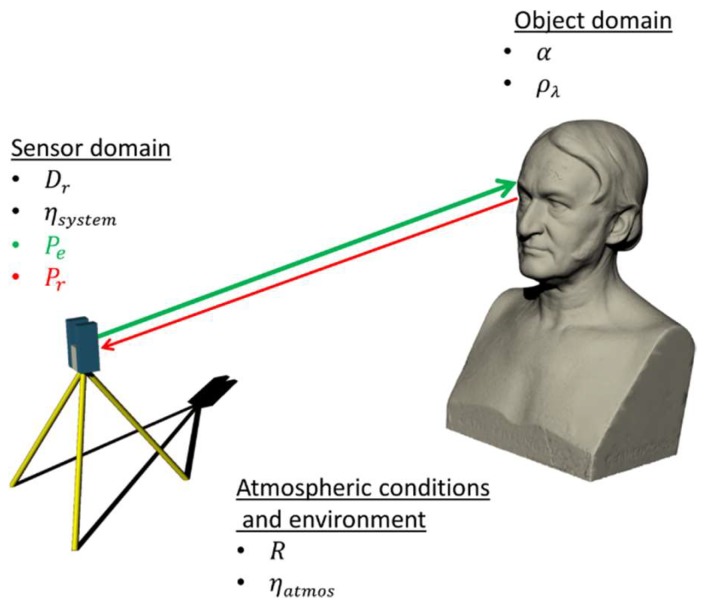
Influencing factors of the signal strength of reflectorless rangefinders.

Figure 1 illustrates the scope of all factors which can be grouped in the following three main influencing factors:

(i) Sensor domain: In which an optical signal with the power *P_e_* is emitted by a laser diode that is then deflected to the environment.
(ii) Environment: While travelling through the environment, the signal is subject to deterioration as a consequence of the range between scanner and an object’s surface as well as the atmospheric transmission coefficient *η_atmos_*.
(iii) Object domain: On the object’s surface the signal is additionally weakened in dependence to the local quotient of reflection *ρ_λ_* as well as the angle of incidence *α*. Another source which causes a loss of signal strength is provoked by the light collector of the scanner, as well as its deflection unit, that initiates the scanning process. The last two mentioned influences are summarised by *η_system_* and are individual characteristics of the applied scanner that, hence, fall into to the sensor domain.

A restriction of the methodology proposed by Wujanz et al. [18] is related to the necessity of repeatedly capturing ranges, which requires a deactivation of the scanner’s deflection unit. This functionality is usually prohibited due to health and safety regulations, especially regarding eye safety. Hence, the first research question of this contribution is dedicated to the search for alternative approaches in order to experimentally determine the stochastic properties of a terrestrial laser scanner’s rangefinder. Two novel methods are proposed in Section 2 in order to derive intensity-based stochastic models based on capturing geometric primitives in the form of planar panels.

The second research focus is set on the transferability of intensity-based stochastic models and tries to clarify if these models are universally valid for all rangefinder types since, thus far, only TLS with phase-shift rangefinders were investigated. The basic principle of phase-shift rangefinders is explained, e.g., by Kahmen and Faig [25] (p. 151ff) and Vosselman and Maas [26] (p. 5ff). Lichti [4] presented an extensive investigation of a laser scanner that applies this rangefinder technology. The transferability of intensity-based stochastic models is subject of Section 3 where laser scanners with rangefinders that apply the impulse method as explained, e.g., by Kahmen and Faig [25] (p. 150), which is often also referred to in the literature as the time-of-flight-principle (tof), e.g., by Vosselman and Maas [26] (pp. 3ff), and hybrid rangefinders specified by Maar and Zogg [27] that combine impulse and phase-shift approaches are analysed. A discussion about the findings of the contribution at hand can be found in Section 4.

## 2. Determination of Intensity-Based Stochastic Models Based on Point Clouds

**Figure 2:**
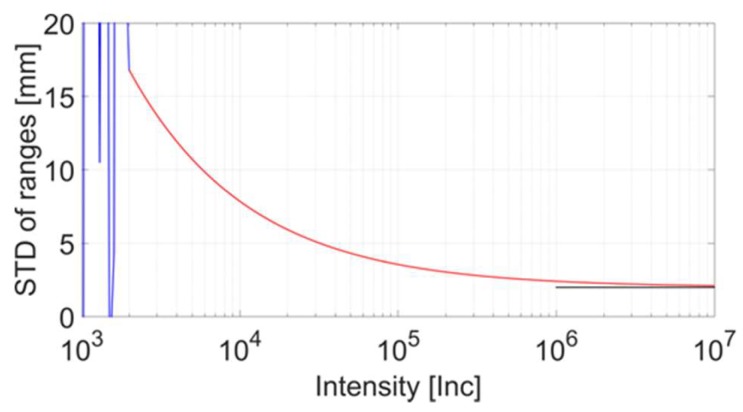
Characteristic ranges of intensity values versus range noise illustrated for a fictitious laser scanner.

This section presents two methods for determining intensity-based stochastic models using planes. The planarity of the samples used is below the measuring precision of the applied laser scanner’s rangefinder and, therefore, does not affect the result. If this assumption does not hold, the imperfection of the applied plane would directly distort the computed stochastic measures. An important issue related to the generation of intensity-based stochastic models is that a potentially large range of intensity values should be acquired. This is of great importance in order to reliably capture the characteristic operation of the scanner’s stochastic behaviour. The run that describes the stochastic model is mainly influenced by the characteristics of the receiving photo diode as pointed out by Vosselmann and Maas [26] (p. 14) and Mettenleiter et al. [28] (p. 51ff) that derives the range between the scanner and object, as well as the signal’s strength based on the received signal. Figure 2 shows the relevant intensity ranges on the abscissa axis and the associated measurement noise on the ordinate for a fictitious TLS. The red values indicate the usable range of the signal for the determination of an intensity-based stochastic model. The red curve approaches asymptotically to a black line, which shows the resolution of the rangefinder at high intensity values. In other words, the model does not allow drawing conclusions below the resolution of the rangefinder, since the measurements captured in this intensity region randomly differ by the amount of the resolution. As the intensity decreases, the noise increases and finally reaches the blue jagged area, in which correct range measurement is no longer possible. This region is caused by electronic noise yielding arbitrary ranges and consequently unpredictable range noise. Therefore, measurements captured in this region are consequently not considered for further processing.

### 2.1. Interpretation of Residuals as Rangefinder Stochastics

The general idea to interpret the residuals that emerge between a planar target and acquired observations as stochastic properties of a reflectorless rangefinder is, per se, not a novel idea (see, e.g., Böhler et al. [10]). Ozendi et al. [29] applied this approach, among others, and set the range noise of a TLS in relation to intensity, object distance, as well as the angle of incidence. The latter aspect has to be seen quite critical as the noise of a captured range is correlated to the direction from where it has been observed. Since the residuals of a plane are bound to its normal direction an increasing rotation of a planar target in relation to a TLS would decrease the magnitude of the resulting residuals. Soudarissanane et al. [30] avoid this falsifying impact by considering the observational direction for every point.

**Figure 3:**
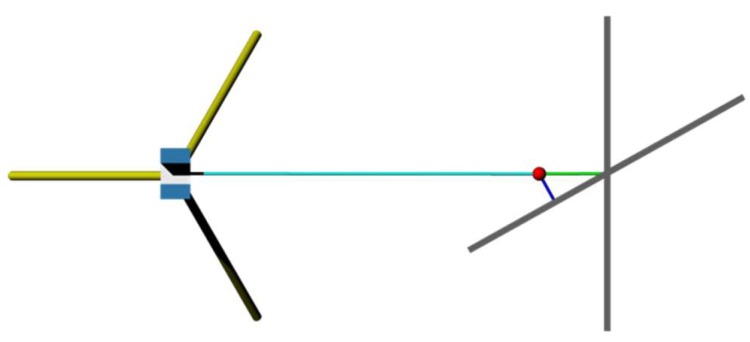
Impact of varying incidence angles onto the residuals.

Figure 3 illustrates the aforementioned circumstance where a distance to a plane is observed under two incidence angles. Note that for illustrative reasons the range noise is assumed to be equal for both scenarios. The resulting point is highlighted by a red circle. It is obvious that the green tinted residual that runs parallel to the observational direction (highlighted by a cyan coloured line), is larger than the blue one which is associated to the face normal of the tilted plane. In essence, this means that the derived range noise is dependent on the orientation of the observed plane. Lambertus et al. [31] noticed and verified this effect within laborious experiments conducted with a time-of-flight scanner, specifically the Leica ScanStation C10. Furthermore Lambertus et al. [31] adapted the idea of linking the signal strength to the arising noise within point clouds. Interestingly, the results show similar characteristics to the findings of Wujanz et al. [18], who applied this to the data from a phase-shift scanner.

A vital aspect which has not received adequate consideration in the context of stochastic modelling of reflectorless rangefinders is the validity of the outcome. This issue will be of particular interest in Section 2.3 where results generated by repeated range measurements are compared against outcomes computed based on the two introduced procedures presented in this subsection as well as Section 2.2. The first experiment included data acquisition of four planar samples with sizes of 50 by 50 cm at different ranges with a Zoller + Fröhlich IMAGER 5006h (Zoller & Fröhlich GmbH, , Wangen im Allgäu, Germany). A GOM ATOS I (GOM GmbH, Brunswick, Germany) structured light scanner was used to verify the planarity of the samples. This adds up to ~0.1 mm and, hence, falls into the range of the applied scanner’s resolution [32]. Every sample features different radiometric properties ranging from black to white. By this, a large spectrum of different intensity values should emerge based on a comparably small set of object distances.

**Figure 4:**
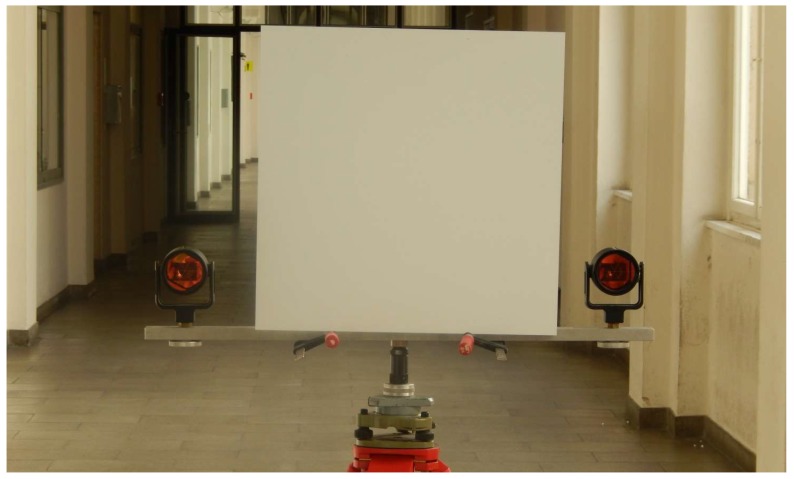
Mount with planar sample.

In total, eight viewpoints with ranges between 5 and 56 m in approximately 6 m increments were observed. At every position four radiometrically different samples were recorded. In order to ensure incidence angles that are nearly parallel to the face normal of a planar sample a mount has been used that is illustrated in Figure 4. The alignment of the mount and, hence, the sample is conducted by using a total station that is fixed to the scanner’s tripod under forced centring and two prisms on the mount. Data acquisition was performed under fixed settings with the resolution setting “ultra high” corresponding to an angular increment of 0.009° and the quality setting “high”. A disadvantage of applying fixed angular settings is that the point sampling captured at different object ranges varies notably. However, this was necessary due to the fact that the sampling rate for Z + F scanners is bound to the aforementioned settings as explained by Mettenleiter et al. [28] (p. 66). Altering these settings would, in turn, change the measurement rate of the rangefinder and thus yield in a notable change of the range noise. Ultimately, a mixture of various noise levels would lead to an inhomogeneous basis for stochastic modelling.

**Figure 5:**
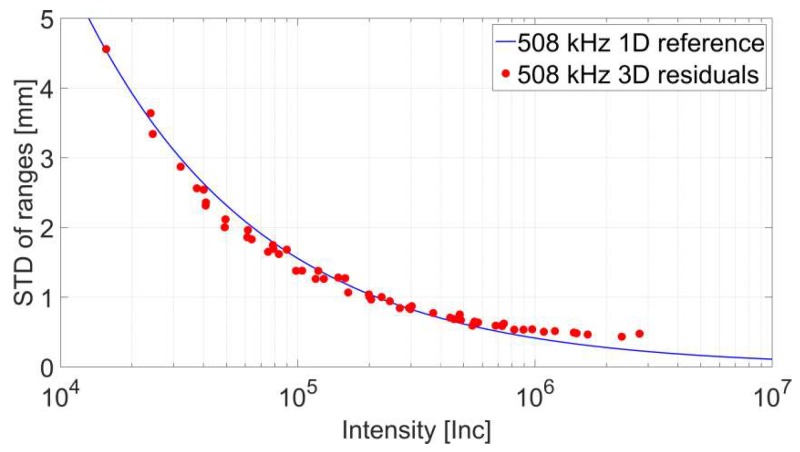
Stochastic model of the reference vs. results from the 3D residuals procedure.

In total 64 datasets were captured. By deploying a least squares adjustment the unknown plane parameters and, finally, the residuals **v** were determined. In contrast to Wujanz et al. [18] all observations received equal weights. Figure 5 depicts the computed results that are highlighted by red circles. The standard deviation of ranges is graphed on the vertical axis while the raw intensity values can be found on the horizontal axis. The blue line represents the stochastic reference model generated by repeated range observations for a sampling rate of 508 kHz [18]. While the computed stochastic measures largely comply with the reference run, differences in the magnitude of up to 0.17 mm can be spotted for higher intensity values. The reason for this characteristic is likely to be caused by larger changes of the incidence angle for panels acquired at close range as opposed to large object distances. A solution to this problem could be to restrict the incidence angle of points on a sample to a certain range.

### 2.2. Rangefinder Stochastics Based on Quasi-Ranges

A disadvantage of the procedure described in the previous subsection is linked to the dependence of the computed range noise to the angle encoders. In order to avoid this potentially falsifying effect, an alternative method to generate stochastic measures for a TLS rangefinder is proposed. The general idea behind this approach is to compute the range stochastics solely based on the recorded distances. In order to achieve this, the control software of the applied TLS has to offer an export function that includes the originally-observed polar elements direction *φ*, tilt angle *θ*, and range *ρ*, as well as the recorded raw intensity. For the applied scanner’s control software *LaserControl* an export option that includes all of the aforementioned elements is available. Therefore, the *asc*-suffix has to be chosen (*Range/intensity as ASCII* (*uncalibrated*)). Instead of using point clouds that describe the entire surface of the planar test sample, patches, of varying size are observed.

**Figure 6:**
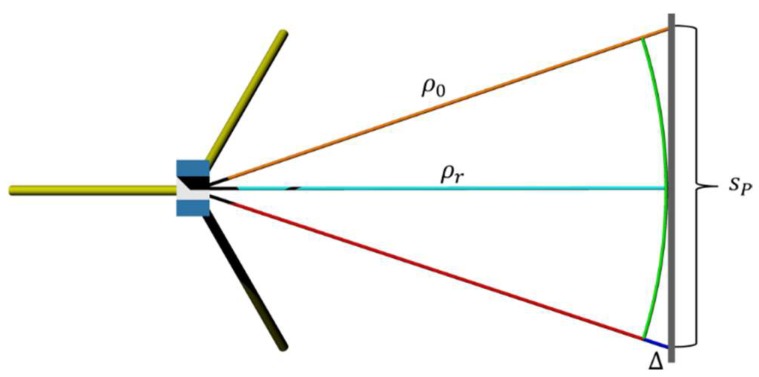
Influence of the direction of observation onto the object distance.

The reason for this is illustrated in Figure 6 where a planar sample with a length of *s_p_*, represented by a grey line on the right, is captured by a TLS. A representative range *ρ_r_* is tinted in cyan for which stochastic properties should be computed. The observed range *ρ_o_* is represented by the orange line. The green coloured semi-circle has a radius of *ρ_r_*. It can be seen that the discrepancy Δ between semi-circle and planar sample, highlighted by a blue line, increases towards the boundaries of the sample. Due to this effect differently sized regions have to be acquired in dependence to the object distance and the resolution/precision of the applied TLS’ rangefinder. Note that this geometrically provoked effect decreases with increasing object distance. However, if the entire sampled area would be considered, the resulting stochastic characteristics would increase as a consequence. Due to the fact that all captured ranges for a certain configuration are slightly different, the term “quasi-range” is used throughout the remainder of the paper.

**Figure 7:**
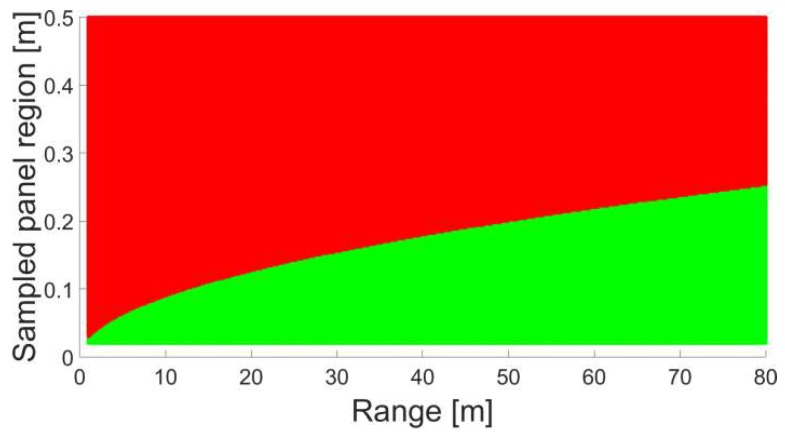
Size of the sampled panel region in dependence to the object range.

The mentioned problem caused by the proposed survey configuration can be tackled by scanning only a restricted region on the sample. In order to determine the expansion of this patch in dependence to the object distance some numerical investigations were undertaken. Therefore, the discrepancy Δ is computed by:

(2) $$\Delta =\sqrt{\rho_{r}^{2}+{\left(\frac{S_{P}}{2}\right)}^{2}}-\rho_{r}$$

where *S_P_* denotes the extent of the sampled panel region. Discrepancies Δ larger than the range resolution of the applied laser scanner are assumed to cause erroneous results and are, hence, omitted. For the applied TLS the resolution sums up to ~0.1 mm [32]. Figure 7 illustrates the computed results where the range between scanner and sample is graphed on the horizontal axis. The vertical axis features the maximum extent of the area under consideration. Red tinted areas in the figure represent configurations where Δ exceeds the resolution of the scanner. As a consequence the corresponding range stochastics would be falsified. Green regions highlight combinations that are suitable to compute the stochastic behaviour of reflectorless rangefinders. It can be seen that the acquirable area on the panel increases with rising range.

**Figure 8:**
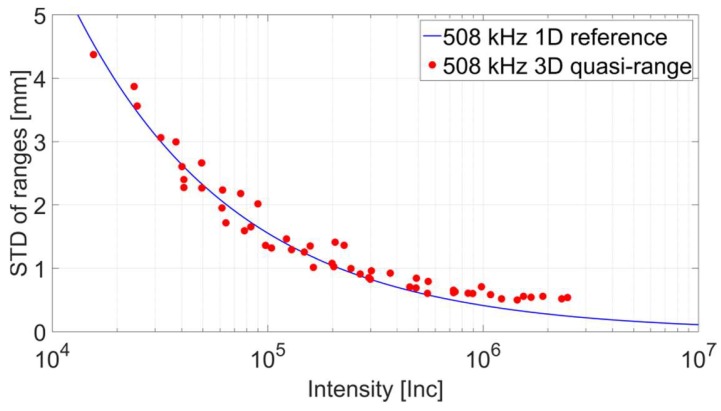
Stochastic model of the reference vs. results from the quasi-range procedure.

Based on the considerations depicted in Figure 7, a stochastic model has been generated by deploying the quasi-range procedure. Therefore, the same captured data has been used yet new input files were exported and cut to the corresponding maximum extent of the sampled panel region. Based on this information the standard deviation for ranges has been computed and graphed against the average intensity as illustrated in Figure 8. Again the reference is highlighted by a blue line while red circles denote the outcome of the quasi-range method. Similar to the findings presented in the previous subsection both datasets coincide quite well for lower intensities yet drift notably apart with increasing strength of the recorded signal. In comparison to Figure 5 the difference between reference and results generated based on the quasi-range method is notably larger. In addition, the run of the computed points is a lot noisier than the outcome presented in Section 2.1. Thus, the conclusion can be drawn that the quasi-range method is quite delicate against an imperfection of the survey configuration in the form of a relative rotation between the scanner and the sample.

### 2.3.Evaluation of the Proposed Procedures

Based on the generated results depicted in Figure 5 and Figure 8 two intensity-based stochastic models were derived. Therefore the following functional model:

(3) $$\sigma_{\rho }=a\cdot {lnc}^{b}+c$$

was used. The three unknown parameters *a*, *b*, and *c* can be determined from a least squares adjustment considering the raw intensities *Inc.* as observations and the values for the standard deviations *σ_ρ_* as fixed parameters. Thus, Equation (3) will later on form the stochastic model for the precision of ranges *σ_ρ_*. Table 1 contains a comparison among a reference model against the parameters which were derived in Section 2.1 and Section 2.2. The reference stems from Wujanz et al. [18] and was captured by repeated observation of ranges at a sampling rate of 508 kHz. Note that it is vital to introduce raw intensity values into the estimated functions that report the precision for ranges in metres.

In order to prove the validity of the generated stochastic model a procedure suggested by Wujanz et al. [18] is used. The basic concept is in accordance to the overall model test of an adjustment as explained e.g., by Teunissen [33] (p. 93) which decides if both, the functional and the stochastic model, are appropriate but also if potential outliers are within a given set of data. In order to validate the outcome of the adjustment, the following information is required:

- observations that were captured with the TLS under investigation;
- a functional model that is capable to represent the recorded data; and
- a stochastic model that describes the precision of the measurements.

The observations that serve as input of the procedure are scans of planar shapes captured under randomly chosen survey configuration and radiometric properties. It is vital that the measurements are independent from the datasets that were used to derive the stochastic model. Under the assumption that a suitable functional model has been chosen and that no or negligibly few outliers are among the observations, it can be checked if the proposed stochastic model is appropriate. Therefore the empirical reference standard deviation:

(3) $$s_{0}=\sqrt{\frac{v^{T}P^{v}}{f}}=\sqrt{\frac{\Omega }{f}}$$

is computed and compared against the standard deviation of the unit weight *σ*_0_, which is also referred to as theoretical reference standard deviation. The sum of weighted squared residuals Ω can also be expressed in matrix notation where **v** denotes the residual vector and **P** the weight matrix of the observations. The value *f* describes the degrees of freedom of the stated adjustment problem. The stochastic model is regarded as being appropriate if the empirical standard deviation *s*_0_ falls into the interval of 0.7 < *s*_0_ < 1.3 under the assumption that the standard deviation of the unit weight *σ*_0_ was set to 1 as proposed, e.g., by Müller et al. [34] (p. 345).

**Figure 9:**
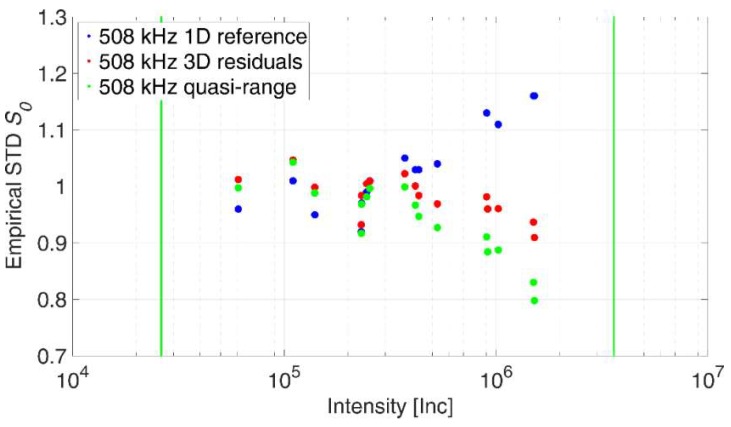
Empirical standard deviations based on the reference stochastic model, as well as the novel approach.

Figure 9 illustrates the results of the verification process. Therefore, 16 datasets from Wujanz et al. [18] were used featuring planar panels that were captured under random survey configurations and varying radiometric properties. The corresponding data was then processed in a least squares adjustment using three different stochastic models yielding in different weights for individual ranges and, consequently, different empirical standard deviations *s*_0_. The horizontal axis shows intensity values on a logarithmic scale. The vertical green lines highlight the smallest and largest intensity values that were captured in the experiments for the generation of the stochastic models. The vertical axis graphs the empirical standard deviations *s*_0_ that stem from the plane adjustments.

The first model was generated based on repetitive range measurements according to Wujanz et al. [18] while the corresponding results are depicted by blue circles. For the red circles the stochastic model generated in Section 2.1 was used, while the green circles stem from Section 2.2. All green and red circles fall into the specified interval of 0.7 < *s*_0_ < 1.3. Hence, this outcome verifies the two suggested procedures for the generation of intensity-based stochastic models based on capturing 3D-point clouds instead of repeated range measurements. Note that the empirical standard deviations drift apart for higher intensities while a higher degree of conformity was apparent for the remaining parts.

## 3. Intensity-Based Stochastic Models for TLS with Impulse and Hybrid Rangefinders

While earlier research by Wujanz et al. [18,19] focussed on stochastic modelling of phase-based laser scanners, the question arose if it is also possible to determine intensity-based stochastic models for scanners with impulse-rangefinders or TLS that apply hybrid approaches, as described by Maar and Zogg [27]. A scanner that falls into the first mentioned category is the Riegl VZ-400i (RIEGL Laser Measurement Systems GmbH, Horn, Austria) that will be under investigation in Section 3.1. Leica’s P40 is a TLS whose rangefinders can be associated with the second category. Table 2 summarises some specifications of the applied laser scanners in this study. Note that the angular values specified for the Riegl in Table 2 denote the corresponding resolution and not their precision.

### 3.1. Stochastic Modelling of an Impulse Scanner

**Figure 10:**
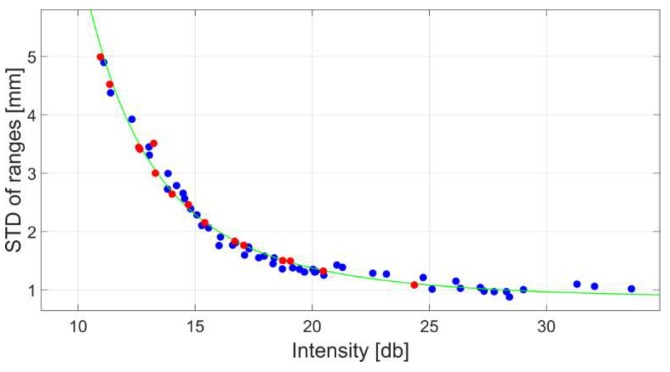
Results of the experiment (blue circles) and generated intensity-based stochastic model (green line). Red circles indicate independent control measurements.

In this section a Riegl VZ-400i [35] is under investigation. To analyse the stochastic properties of the scanner the rangefinder was operated at 1.2 MHz yielding in “multiple time around” zone 1 (MTA). This MTA 1 zone signifies the circumstance that only one emitted pulse is travelling between the scanner and an object that is not more than 125 m away. For longer ranges ambiguities occur since several pulses travel on the very same optical path. However, integrated software in the scanner is capable to resolve ambiguities. In order to derive the intensity-based stochastic model for the scanner of interest the procedure suggested in Section 2.1 was used. For that, eight panels with varying radiometric properties were captured at ranges between 10 and 116 m. Every captured panel yields to one blue circle in Figure 10 based on the corresponding mean intensity, as graphed on the horizontal axis, and the precision of range which is plotted on the vertical axis. Red circles highlight the independent control measurements under random survey configuration that were used to verify the validity of the computed stochastic model. Note that the horizontal axis is not divided on a logarithmic scale in contrast to the results presented in Section 2. However, the characteristics of the run are comparable to the results presented in Section 2 since the intensity values are given in a decibel format, which is already of logarithmic nature. The raw intensity values were exported using the scanner control software provided by the manufacturer.

To derive an intensity-based stochastic model, the functional relationship described in Equation (3) was chosen. After the adjustment the stochastic model follows the parameters gathered in Table 3. The resulting curve is illustrated by a green line in Figure 10.

For the numerical verification of the computed stochastic model additional 16 scans were captured under randomly chosen survey configurations. Table 4 summarises the configuration where the first column contains the range between the origin of the scanner’s coordinate system to the centre of the observed plane. The second column contains the incidence angle of the beam at the centre of the panel while the average intensity is given in the third column.

**Figure 11:**
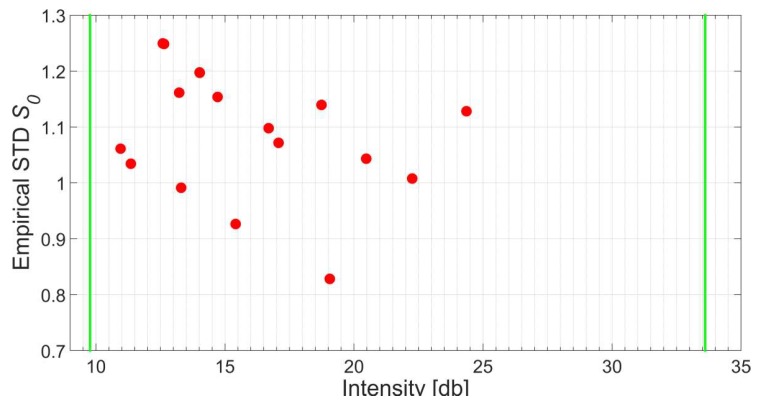
Empirical standard deviations of adjusted planes captured under different survey configurations. The two green vertical lines indicate the smallest and largest intensity values that stem from the experiments to generate the intensity-based stochastic model.

The results are depicted in Figure 11 where the empirical standard deviation *s*_0_ for every panel after the plane adjustment is graphed on the vertical axis while the horizontal axis features the mean signal strength of the corresponding points. Since all values *s*_0_ fall into the specified boundaries 0.7 < *s*_0_ < 1.3 it can be concluded that the generated intensity-based stochastic model is appropriate to describe the random characteristics of the scanner under investigation. In addition, the suggested procedure is generally capable to model rangefinders and, consequently, scanners that deploy the impulse method.

### 3.2. A Stochastic Model for a Hybrid TLS

This section analyses the stochastic properties of a Leica ScanStation P40 [36] (Leica Geosystems, Heerbrugg, Switzerland). In contrast to all other TLS investigated by Wujanz et al. [18,19] and in Section 3.1, the scanner control software provided by the manufacturer does not allow to export raw intensity values. After correspondence with Mr. Tüxsen from Leica Geosystems AG, the functional relationship was released that allows retrieving the original intensity values. In order to compute the stochastic model for this instrument two series of experiments were conducted since the scanner under investigation allows capturing data in two modes, namely a speed mode with a range of up to 120 m, as well as a range mode with maximum ranges of 270 m [37]. In the first experiment carried out in speed mode, eight panels with varying radiometric properties were captured at ranges between 12 and 116 m yielding in 66 datasets. In the second experiment the data was captured in range mode and focussed on ranges between 47 and 181 m leading to 64 samples.

**Figure 12:**
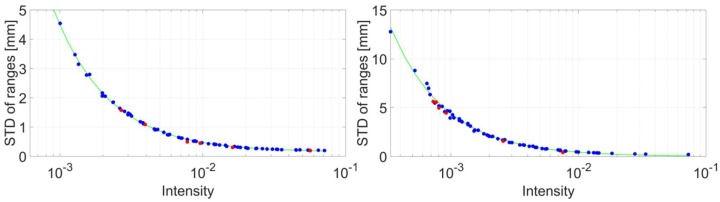
Results of the experiment (blue circles) and generated intensity-based stochastic model (green line) for speed mode (**left**) and range mode (**right**). Red circles highlight control measurements that were used to verify the computed stochastic model.Again, it was assumed that the intensity-based stochastic model follows the functional relationship described in Equation (3). The resulting parameters after the adjustment are gathered in Table 5. Note that the third parameter *c* was not of significance for the range mode.

Figure 12 illustrates the results of the experiments. The horizontal axis is of a logarithmic scale depicting intensity values, while the vertical axis graphs the range precision in millimetres. Blue circles denote data that was used to derive the stochastic model; the green lines represent the computed stochastic model while the red dots signify independent measurements under random survey configuration in order to verify the validity of the computed model. The left part shows the outcome based on data captured in speed mode. A systematic run is apparent that supports the assumption that intensity-based stochastic models are suitable to model the precision of this laser scanner. Another first aspect that speaks for its validity is the proximity of the red circles that denote the control measurements. The very same arguments apply to the right part of the figure that show corresponding results for the measurements captured in speed mode.

For the verification of the stochastic model 13 additional scans were captured; seven under use of the speed mode and six by using the range mode. The resulting survey configuration can be found in Table 6, while the structure of the content is identical to Table 5. Note that no unit is specified for the given intensity values.

**Figure 13:**
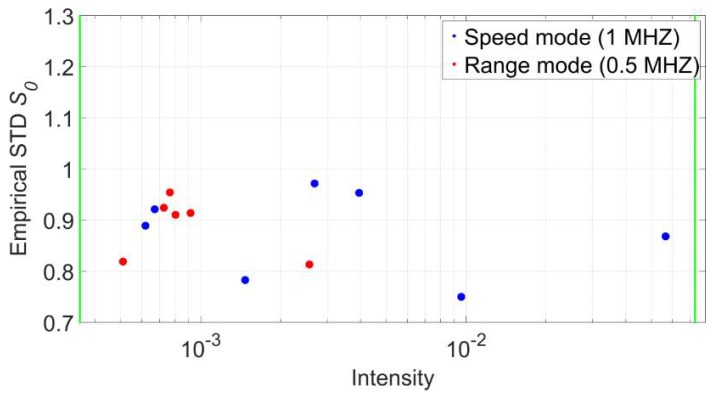
Empirical standard deviations of adjusted planes captured under different survey configurations.

Based on the aforementioned measurements and the general concept of the overall model test [33] (p. 93) the computed stochastic models for both the speed, as well as the range mode, were verified. Figure 13 illustrates the results. The horizontal axis features the mean intensity of the corresponding points while the vertical axis contains the empirical standard deviation *s*_0_ for every panel after the plane adjustment. Just as in Figure 11 the two green vertical lines mark the smallest and largest intensity values from the experiments that were used to generate the intensity-based stochastic model. All values fall into the specified boundaries of 0.7 < *s*_0_ < 1.3 which allows drawing the conclusion that both computed stochastic models are adequate to model the stochastic characteristics of the scanner. All empirical standard deviations are smaller than 1, which means that the generated stochastic model releases values that are slightly too pessimistic by trend. Thitherto, no explanation for this characteristic was found and, hence, will be addressed in future investigations. 

## 4. Conclusions

This article demonstrates the circumstance, at first, that intensity values captured by terrestrial laser scanners inherently consider influences provoked by the survey configuration, the radiometric properties of a scanned surface, meteorological effects, as well as scanner-specific characteristics. Hence, intensity values are, per se, an appropriate input for all-embracing stochastic models of reflectorless rangefinders deployed, e.g., in terrestrial laser scanners. The suggested methodology does not require individual consideration of the influencing parameters—a course of action that is doomed to fail, as shown in Section 1. A clear disadvantage of the initial procedure proposed by Wujanz et al. [18] is its dependence to operate the rangefinder of the scanner under investigation in 1D-mode, a functionality that is usually prohibited due to health and safety regulations. In order to allow the generation of intensity-based stochastic models with TLS in normal operation (3D-mode) two novel approaches were suggested that are based on capturing planar shapes utilising 3D-point clouds. The final research question focussed on the universal validity of intensity-based stochastic models for all rangefinder types. While previous research by Wujanz et al. [18,19] exclusively investigated laser scanners that incorporate phase-based rangefinders, a Riegl VZ-400i impulse scanner, as well as a Leica ScanStation P40, that applies a hybrid rangefinder strategy, were investigated in this contribution. Since the stochastic characteristics of these laser scanners can also be described by intensity-based stochastic models, their generality was assured. In order to allow spreading the idea of intensity-based stochastic models into practice, it is desirable that some manufacturers offer the possibility to export unprocessed intensity values—a rather simple demand which, however, is only offered by very few companies. Prospective research will focus on ambient influences onto terrestrial laser scanners, as well as on configurations that yield the systematic biases of the rangefinder.

## Figures and Tables

**Table 1:** Comparison of several stochastic models.

Stochastic Model from	$\hat{a}$	$\hat{b}$	$\hat{c}$
Reference	1.1742	−0.5756	---
Residuals	4.1910	−0.7145	0.0003
Quasi-ranges	13.7781	−0.8276	0.0005

**Table 2:** Specification of the laser scanners under investigation.

	Riegl VZ-400i	Leica ScanStation P40
Range [m]	800	270
Angular resolution, accuracy (hz; vt)	2.5″; 1.8″	8″; 8″
Range noise	3 mm (1*σ*) at 100 m	0.5 mm rms at 50 m
Wavelength	Near infrared	1550 nm

**Table 3:** Parameters of the stochastic model for the rangefinder of a Riegl VZ-400i operated in MTA 1.

Sampling Rate (MHz)	$\hat{a}$	$\hat{b}$	$\hat{c}$
1.2	17.5390	−3.4689	0.0008

**Table 4:** Survey configuration for the verification of the computed stochastic model.

Range (m)	Incidence Angle (°)	Intensity (db)
20.974	27.8	24.36
20.977	34.2	18.74
20.988	56.4	17.07
20.989	57.1	22.26
42.121	28.8	14.71
42.123	30.7	20.47
42.128	52.9	19.06
42.140	53.6	13.23
62.645	21.8	12.59
62.651	45.9	10.95
62.651	21.2	16.69
62.663	46.1	15.41
101.091	44.8	13.30
101.091	24.1	14.02
101.099	17.7	12.63
101.114	44.6	11.35

**Table 5:** Stochastic model for the rangefinder of a Leica ScanStation P40 operated in speed and range mode.

Rangefinder Mode | Sampling Rate (MHz)	$\hat{a}$	$\hat{b}$	$\hat{c}$
Speed | 1.0	1.904 × 10^−6^	−1.1196	0.0001
Range | 0.5	4.072 × 10^−6^	−1.011	---

**Table 6:** Survey configuration for the verification of the computed stochastic models.

Rangefinder Mode	Range (m)	Incidence Angle (°)	Intensity
Speed	12.681	15.1	0.0566
Speed	23.719	25.0	0.0096
Speed	61.202	7.3	0.0027
Speed	76.022	42.5	0.0015
Speed	91.473	5.5	0.0040
Speed	105.730	19.9	0.0006
Speed	116.415	9.8	0.0007
Range	105.712	13.4	0.0026
Range	127.089	17.4	0.0008
Range	138.355	20.7	0.0009
Range	149.715	19.8	0.0008
Range	163.130	25.8	0.0007
Range	180.638	36.5	0.0005
